# Genotoxicity reduction in bagasse waste of sugar industry by earthworm technology

**DOI:** 10.1186/s40064-016-2882-1

**Published:** 2016-07-27

**Authors:** Sartaj Ahmad Bhat, Jaswinder Singh, Adarsh Pal Vig

**Affiliations:** 1 Department of Botanical and Environmental Sciences, Guru Nanak Dev University, Amritsar, Punjab 143005 India; 2 PG Department of Zoology, Khalsa College, Amritsar, Punjab India

**Keywords:** *Allium cepa* aberration assay, *Eisenia fetida*, Industrial waste, Toxicity, Vermicomposting

## Abstract

The aim of the present study was to assess the genotoxicity reduction in post vermicompost feed mixtures of bagasse (B) waste using earthworm *Eisenia fetida*. The genotoxicity of bagasse waste was determined by using *Allium cepa* root chromosomal aberration assay. Bagasse was amended with cattle dung in different proportions [0:100 (B_0_) 25:75 (B_25_), 50:50 (B_50_), 75:25 (B_75_) and 100:0 (B_100_)] on dry weight basis. Genotoxic effects of initial and post vermicompost bagasse extracts were analysed on the root tips cells of *Allium cepa*. Root length and mitotic index (MI) was found to be increased in post vermicompost extracts when compared to initial bagasse waste. The maximum percent increase of root length was observed in the B_50_ bagasse extract (96.60 %) and the maximum MI was observed in B_100_ mixture (14.20 ± 0.60) 6 h treatment which was similar to the control. Genotoxicity analysis of post vermicompost extracts of bagasse revealed a 21–44 % decline in the aberration frequencies and the maximum reduction was found in B_75_ extract (44.50 %). The increase in root length and mitotic index, as well as decrease in chromosomal aberrations indicates that *E. fetida* has the ability to reduce the genotoxicity of the bagasse waste.

## Background

Enormous generation of industrial solid waste is a major environmental problem in the whole world. The improper disposal of these wastes can degrade the environment and affect the human health. The wastes generated from sugar industrial wastes are pressmud, bagasse, sugar beet mud and pulp (Bhat et al. 2014, 2015a, b). The management of these wastes are important in controlling the environmental pollution and contamination. Earthworm technology (vermitechnology) has the ability to reduce the toxicity of industrial wastes (Rezende et al. 2014). It involves degradation of organic waste into stable material by combined activities between earthworms and microbes living in their gut (Dominguez and Edwards 2011; Bhat et al. 2013; Haynes and Zhou 2016). Use of earthworms for toxicity reduction in industrial wastes has been used by many researchers (Jain et al. 2004; Srivastava et al. 2005; Bhat et al. 2014, 2015b). The chlorogocyte cells and the intestinal microflora of earthworms have the capability to decrease the genotoxicity of industrial wastes (Srivastava et al. 2005). *Allium cepa* root chromosomal aberration assay is widely used as a sensitive test in monitoring of waste genotoxicity (Rank 2003; Leme and Marin-Morales 2009). For monitoring environmental pollutants, this sensitive and stable test has also been adopted by the International Program on Plant Bioassay (IPPB; Ma 1999). The different endpoints of *Allium cepa* root chromosomal aberration assay used for assessment of genotoxicity in environment are mitotic index, chromosome aberrations, nuclear abnormalities and micronucleus (Leme and Marin-Morales 2009). Several researchers have used *Allium cepa* root chromosomal aberration assay in genotoxicity assessment of industrial wastes/effluents such as paint and textile industrial effluent (Samuel et al. 2010), electronic waste leachate (Bakare et al. 2012), tannery effluent (Masood and Malik 2013), pressmud sludge (Bhat et al. 2014), sugar beet mud (Bhat et al. 2015b), domestic sewage sludge (Mazzeo et al. 2015) etc. In the earlier study, Bhat et al. (2015a), bagasse waste amended with cattle dung was managed by vermicomposting process. The growth parameters of earthworm and physico-chemical analysis of feed mixture were done to know the nutrient content of vermicompost. In continuation of the previous study the vermicompost produced by the earthworm was assessed by genotoxicity test. In the current experiment *A. cepa* root chromosomal aberration assay was performed to evaluate the toxicity/genotoxicity reduction of the bagasse waste before and after vermistabilization.

## Methods

### Earthworm and sugar industrial waste collection

In the present study young non-clitellated *E. fetida* were selected from a stock culture maintained in the vermicomposting unit of the Department of Botanical and Environmental Sciences, Guru Nanak Dev University, Amritsar, India. Cattle dung (CD) was arranged from the local dairy. Bagasse (B) was obtained from Rana Sugars Limited, Amritsar, India.

### Experimental setup

Five proportions with different ratios of B and CD were prepared, namely, 0:100 (B_0_) 25:75 (B_25_), 50:50 (B_50_), 75:25 (B_75_) and 100:0 (B_100_) in plastic trays in triplicates were used for vermicomposting. The vermicomposting process was conducted for 135 days and almost 30 g of the substrate was collected on the first and last day of experiment as described earlier (Bhat et al. 2015a). The collected substrate from each tray was air dried, sieved and stored in polythene bags for genotoxicity analysis.

### *Allium cepa* root chromosomal aberration assay

#### Extract preparation

The pre and post vermicompost samples were prepared according to the French Standard method (Ferrari et al. 1999) i.e. 1:10 (w:v) using double distilled water. The samples were shaken continuously for 24 h and filtered through Whatman filter paper No. 42 and the final extract was analyzed for root growth and genotoxicity studies. The extracts were subjected to 6 and 12 h treatment period to evaluate the frequency of chromosomal aberrations before and after vermicomposting.

#### Root growth test

Onion bulbs were placed on couplin jars containing different pre and post vermicompost extracts. The root length test was performed as a 96 h test (Rank 2003). The extracts were changed every after 48 h. After 96 h of experiment, the onion bulbs were washed in tap water and the best 10 root length of each onion was measured with the help of thread. The mean root length was calculated in centimeters.

#### Genotoxicity test

The genotoxicity of the pre and post vermicompost bagasse extracts was analysed using *A. cepa* root tip cells. The onions were denuded and were grown in coupling jars containing tap water for 24–36 h. The roots (0.5–1 cm) of onion bulbs were then placed in treatment jars containing different extracts of pre and post vermicompost bagasse (0, 25, 50, 75 and 100 %). The exposure time for each pre and post vermicompost extract was 6 and 12 h respectively. After the 3 and 6 h of treatment, the root tips were washed, fixed in farmer’s fluid (1:3, glacial acetic acid:ethanol) for 24 h and stored at cold temperature (4 °C).

#### Preparation of slides

The slides were prepared by hydrolyzing the root tips with 1 N HCl and then squashed in 9:1 ratio of aceto-orcein and 1 N HCl with intermittent heating for 1–2 min. After 25 min in aceto-orcein, the root tips were removed and immersed for 30 s in a drop of 45 % acetic acid. The root tips were then put on a clean slide, covered with a cover slip, squashed by match stick and sealed with a DPX solution. Each slide of the extract was labelled, examined/scored under microscope at magnification of 100× and photographs of normal and aberrant cells were taken. The total number of cells, diving cells and aberrant cells were counted in each pre and post vermicompost extract. Chromosomal abnormalities were scored in 450 cells of each extract. The chromosomal aberrations were classified into physiological (c-mitosis, delayed anaphases, laggard chromosomes, stickiness, vagrant chromosomes) and clastogenic (chromatin bridges and chromosomal breaks). The chromosomal aberrations were represented in percentage and were calculated by the number of aberrant cells as a percentage of total dividing cells for each extract. The cytotoxic activity of the pre and post vermicompost bagasse extracts was evaluated by the mitotic index (MI), through the number of dividing cells as the percentage of a total number of cells analyzed for each concentration.

$${\text{Mitotic}}\;{\text{index}} = \frac{{{\text{Number}}\;{\text{of}}\;{\text{dividing}}\;{\text{cell}}}}{{{\text{total}}\;{\text{number}}\;{\text{of}}\;{\text{cells}}}} \times 100$$

### Statistical methods

The mitotic index and root length were presented as mean ± SE of triplicate experiment and the level of significance was determined by Student’s paired *t* test. The chromosomal aberrations were represented in percentage and the significance level was determined by Chi square test. Minitab version 14.0 was used for Statistical analysis.

## Results and discussion

### Root growth test

**Fig. 1:**
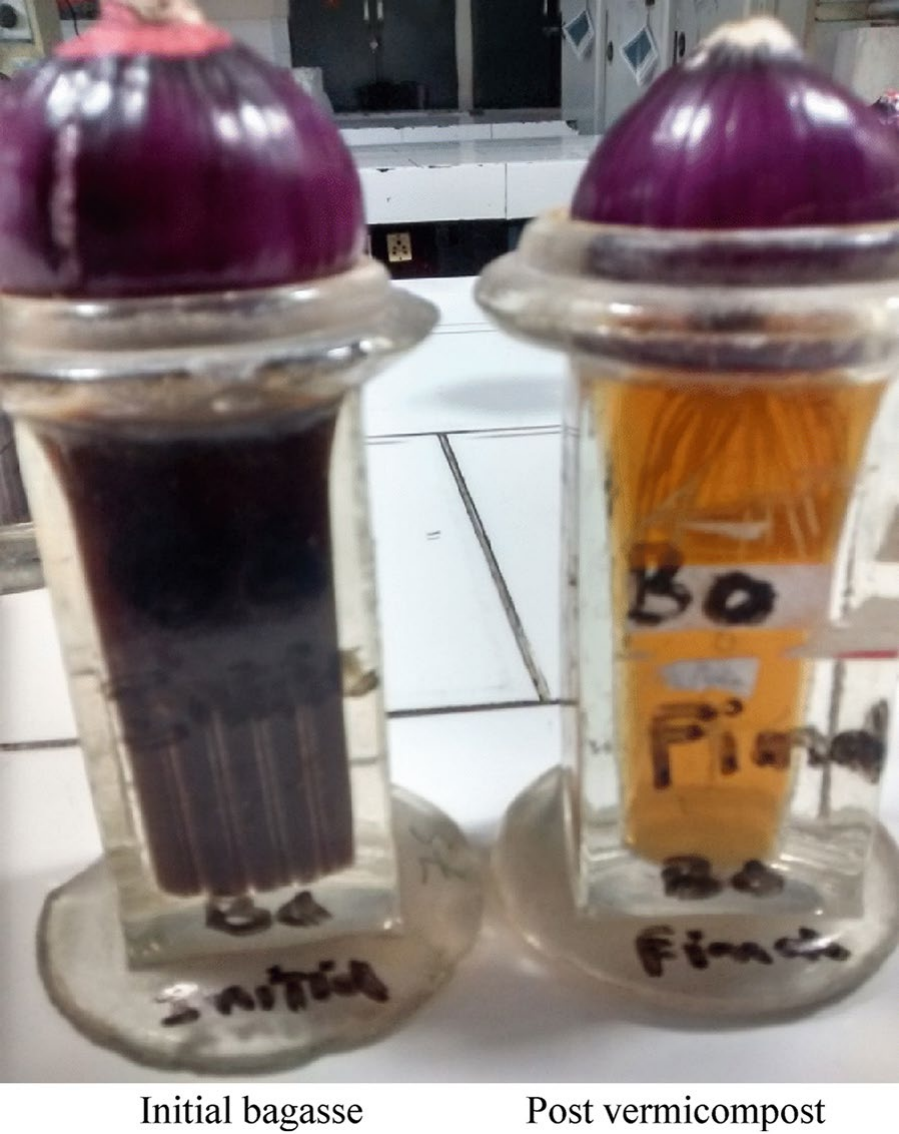
Comparison of initial and post vermicompost extract showing increase in root length and clarity of final filtrate/extract

While preparing extracts from solid samples of initial and post vermicompost samples of all feed mixtures, it was observed that extracts of final vermicomposted samples were showing relatively clear and colorless solutions as compared to initial extracts (dark solutions; Fig. 1). The effects of pre and post vermicompost bagasse extracts on root length of *A. cepa* are shown in Table 1. The initial and final values of bagasse extracts were significantly different (*P* < 0.05). The root length of post vermicompost extracts showed a significant increase in root length as compared to initial bagasse extracts. The maximum percentage increase of 96.60 % in root length was observed in B_50_ extract and the minimum percentage increase of 53.71 % was observed in B_100_ bagasse extract. Decrease of root growth in the initial bagasse extracts can be due to the occurrence of chromosomal aberrations and reduction in mitotic activities. Sumitha and Thoppil (2015) observed that the decrease in root length of plant extracts may be due presence of some substances that impair cell expansion and differentiation of *A. cepa* root tip cells. Reduction of root growth in *A. cepa* root chromosomal aberration assay indicates cytotoxicity and growth retardation (Grant 1999).

**Table 1:** Mean (±SE) root length of *A. cepa* exposed to initial and post vermicompost bagasse (B) extract for 96 h

Concentrations (%)	Initial bagasse waste	Post vermicompost
	Mean root length (cm)	Mean root length (cm)
0 (control)	3.66 ± 0.40	5.46 ± 0.41 (49.18)
25	3.54 ± 0.25	6.76 ± 0.36* (90.96)
50	3.24 ± 0.29	6.37 ± 0.21* (96.60)
75	2.96 ± 0.14	4.55 ± 0.31** (53.71)
100	2.18 ± 0.33	3.40 ± 0.17 (55.96)

The significance level was determined by Student’s paired ‘*t*’ test

* *P* < 0.05; ** *P* < 0.01

Percent change is shown in parenthesis

### Mitotic index and chromosomal aberrations

The effects of initial and post vermicompost bagasse extracts on the MI of *A. cepa* root cells are shown in Table 2. The initial and post values of MI in all bagasse extracts are significantly different (*P* < 0.05). The percentage of MI was significantly increased in the post vermicompost extracts as compared to initial bagasse extract. The maximum percentage of MI was observed in 100 % concentration (14.2 ± 0.60 %) of final vermicompost bagasse extract during 6 h treatment while the minimum percentage MI was observed in 6 h treatment of 100 % bagasse extract (9.38 ± 0.03 %) of initial bagasse extract. The MI was lower at 6 h exposure time than at 12 h. Decrease in MI in the initial feed mixtures inhibits cell division which reflects that the bagasse waste is cytotoxic. The interference in the normal cell cycle leads to decrease in the number of dividing cells which reduces MI (Sharma and Vig 2012). According to El-Ghamery et al. (2000) the mitotic index reduction may be due to the protein synthesis or inhibition of DNA. Bhat et al. (2014, 2015b) also observed a decrease in MI in the initial mixtures of pressmud and sugar beet pulp waste. The results suggested that the initial mixtures of sugar industrial wastes presented cytotoxic effects where as the majority of post vermicomposted feed mixtures did not have any cytotoxic effects. Jain et al. (2004) also observed a decrease in MI in initial mixtures of flyash whereas mitotic index was increased in the post vermicomposted mixtures of flyash. MI in the final vermicomposted mixtures of municipal sludge has also been observed by Srivastava et al. (2005). The MI results show that the vermicomposting of industrial wastes might be beneficial before dumping/landfilling.

**Table 2:** Effect of initial and post vermicompost extract of bagasse (B) on mitotix index of the root meristimatic cells of *A. cepa*

Concentration	Mitotic index in initial bagasse waste^a^	Mitotic Index in post vermicompost^b^
0 % (control)		
6 h	9.57 ± 0.34	11.83 ± 0.87
12 h	10.62 ± 0.41	12.88 ± 0.07*
25 %		
6 h	9.97 ± 0.42	11.05 ± 0.42
12 h	10.74 ± 0.15	11.35 ± 0.22*
50 %		
6 h	10.25 ± 0.53	12.27 ± 0.10*
12 h	11.53 ± 0.25	12.12 ± 0.34*
75 %		
6 h	9.69 ± 0.42	12.45 ± 0.18**
12 h	9.07 ± 0.54	12.12 ± 0.51*
100 %		
6 h	9.38 ± 0.03	14.20 ± 0.60*
12 h	10.17 ± 0.84	11.76 ± 0.58*

The significance level was determined by Student’s ‘*t*’ test

* *P* < 0.05; ** *P* < 0.01

^a^From each group 3802–4791 cells were scored to determine MI

^b^From each group 3180–4661 cells were scored to determine MI

**Fig. 2:**
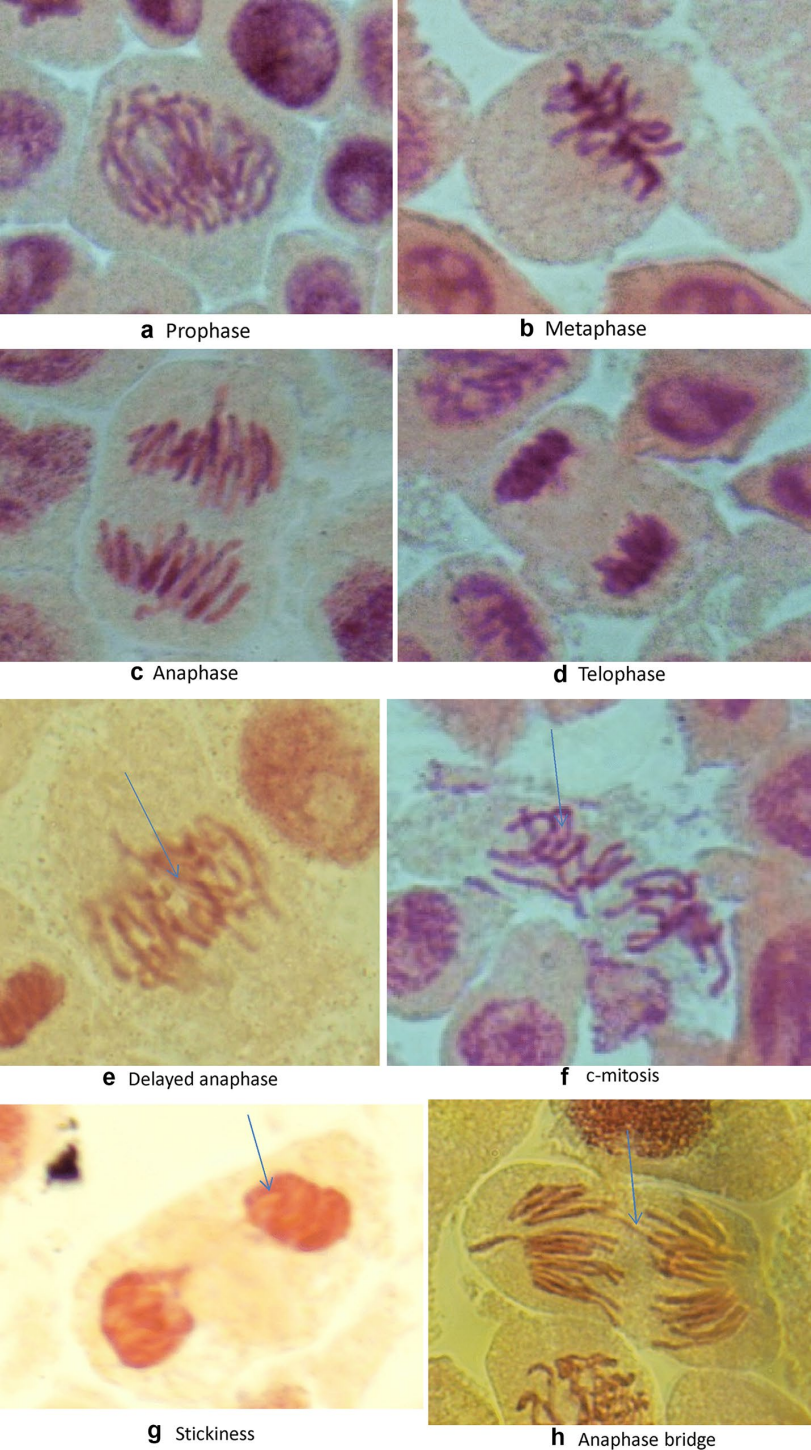
Root tip cells of *Allium cepa* showing normal stages (**a**, **b**, **c**, **d**) and chromosomal aberrations (**e**, **f**, **g**, **h**)

The number and types of chromosome aberrations in the initial and post vermicompost bagasse extracts are summarized in Table 3. In *A. cepa* root chromosomal aberration assay, the major chromosomal aberrations were noted as physiological (delayed anaphase, c-mitosis, laggards, vagrants and stickiness) and clastogenic aberrations (chromosomal bridges and chromosome breaks; Fig. 2). Initial bagasse extracts induced significant (*P* < 0.05) frequencies of chromosomal aberrations whereas post vermicompost bagasse extracts revealed a significant decline (21–44 %) in the chromosome aberrations with maximum reduction in B_75_ feed mixture (44.50 %). The B_100_ extract with 12 h treatment produced maximum chromosomal aberrations (11.33 %). The chromosome aberrations were higher in 12 h exposure time than at 6 h. Delayed anaphases, c-mitosis, stickiness and chromatin bridges were maximum at 100 % of initial bagasse extract whereas 100 % 12 h of post vermicompost bagasse extract showed only minimum (8.88 %) aberrations. Delayed anaphase, c-mitosis, stickiness and chromosome bridges were maximum in all the extracts of bagasse waste.

**Table 3:** Chromosomal aberrations in the root tip cells of *A. cepa* exposed to initial and post vermicompost bagasse extracts

Types of chromosomal aberrations	0 % (control)		25 %		50 %		75 %		100 %	
	No. of Aberrant cells^a^		No. of Aberrant cells^a^		No. of Aberrant cells^a^		No. of Aberrant cells^a^		No. of Aberrant cells^a^	
	6 h	12 h	6 h	12 h	6 h	12 h	6 h	12 h	6 h	12 h
Physiological aberrations (PA)										
C-mitosis	3 (2)	4 (2)	7 (4)	6 (1)	9 (4)	8 (5)	7 (6)	9 (5)	9 (9)	8 (10)
Delayed anaphase	4 (4)	6 (5)	7 (6)	12 (10)	5 (7)	11 (8)	13 (10)	15 (9)	14 (13)	19 (14)
Laggard/s	–	–	–	–	–	–	–	1 (1)	2 (1)	1 (1)
Stickiness	–	1	3 (1)	2 (1)	2 (2)	3 (2)	6 (1)	5 (2)	6 (2)	8 (3)
Vagrant/s	3 (1)	1 (1)	5 (2)	3 (2)	6 (2)	4 (1)	7 (1)	6 (2)	8 (3)	2 (4)
Total PA	10 (7)	12 (8)	22(13)	23 (14)	22 (15)	26 (16)	33 (18)	36 (19)	39 (28)	38 (32)
Clastogenic aberrations (CA)										
Chromatin bridge/s	– (1)	3 (2)	3 (2)	5 (3)	5 (3)	7 (5)	7 (4)	7 (5)	6 (7)	9 (6)
Chromosomal break/s	2	–	–	1	2 (1)	2 (1)	3 (1)	2 (1)	2 (1)	4 (2)
Total CA	2 (1)	3 (2)	3 (2)	6 (3)	7 (4)	9 (6)	10 (5)	9 (6)	8 (8)	13 (8)
Total aberrant cells (PA + CA)	12 (8)	15 (10)	25 (15)	29 (17)	29 (19)	35 (22)	43 (23)	45 (25)	47 (36)	51 (40)
Percent aberration (%)	2.66 (1.77)	3.33 (2.22)	5.55 (3.33)	6.44 (3.77)	6.44 (4.22)	7.77 (4.88)	9.55* (5.11)	10.0 (5.55)	10.44* (8.0*)	11.33* (8.88*)
Percent reduction (%)	33.45	33.33	40.0	41.45	34.47	37.19	41.88	44.50	23.37	21.62

Values of chromosome aberrations in post vermicompost mixtures are shown in parenthesis

The significance level was determined by Chi square test, * *P* < 0.05

^a^Data obtained from 450 cells

Stickiness in the chromosomes may be due to DNA condensation or entanglement of chromatin fibers (Osterberg et al. 1984; Chauhan et al. 1999). Bridges in the chromosome may be due to the stickiness or by dicentric chromosome formation (Jabee et al. 2008). Breaks in chromosomes results from the fragile site breakage (Lukusa and Fryns 2008). Vagrant in chromosomes indicates spindle poisoning (Rank 2003). Bhat et al. (2014) have also observed that the percent aberration was higher (30.8 %) after initial exposure of pressmud sludge, but was reduced to 20.3 % after vermicomposting with *E. fetida*. The chlorogocyte cells and the intestinal microrganisms of *E. fetida* have the ability to detoxify the genotoxicity of industrial wastes (Jain et al. 2004; Srivastava et al. 2005). The present study suggests that initial feed mixtures of bagasse waste showed cytotoxic/genotoxic potential which declines at the end of vermicomposting. The results also revealed that the increase in root length and mitotic index, as well as decrease in chromosome aberrations in the post vermicompost extracts, indicates that *E. fetida* has the ability to detoxify the sugar industrial waste.

## Conclusion

In the present study the results indicate the genotoxicity potential of bagasse waste and also the feasibility of earthworm technology to reduce the toxicity as observed by the results of *A. cepa* root chromosomal aberration assay. Increase in root length and mitotic index as well as decrease in chromosome aberrations in the post vermicompost proportions of bagasse waste indicates that the earthworm *E. fetida* has the ability to reduce the genotoxicity of this waste and the end product can be used safely in agriculture.
